# Ichthyotoxic *Cochlodinium polykrikoides* Induces Mitochondrial Mediated Oxidative Stress and Apoptosis in Rat Liver Hepatocytes 

**Published:** 2013

**Authors:** Jafar Shahraki, Abbasali Motallebi, Marjan Aghvami, Jalal Pourahmad

**Affiliations:** a *School of Pharmacy, Pharmaceutical Sciences Research Center, Shahid Beheshti University of Medical Sciences, Tehran, P.O.Box 14155-6153, Iran. *; b *Department of Pharmacology and Toxicology, Faculty of Pharmacy, Zabol University of Medical Sciences, Zabol, Iran. *; c *Iranian Fisheries Research Organization, Tehran, Iran. *

**Keywords:** *Cochlodinium polykrikoides*, Rat hepatocytes, Oxidative stress, Apoptosis, mitochondria

## Abstract

In this research, we investigated the cytotoxic mechanisms of *Cochlodinium polykrikoides*cell lysate on isolated rat liver hepatocytes.This micro algae is responsible for a severe and widespread harmful algal bloom in the Persian Gulf and Gulf of Oman (2008-2009). Isolated hepatocytes were obtained by collagenase perfusion of Sprague-Dawley rat liver.According to our results, incubation of algal lysate with isolated rat hepatocytescaused hepatocyte membrane lysis, reactive oxygen species (ROS) formation, glutathione depletion, collapse of mitochondrial membrane potential,ATP depletion and increase in ADP/ATP ratio, cytochrome c release in to the hepatocyte cytosol,activation of caspase-3 (final mediator of apoptosis) and appearance of apoptosis phenotype. On the other hand, pre-treatment of antioxidants (*α*-tocopherol succinate and BHT), radical scavengers (mannitol and DMSO), mitochondrial permeability transition (MPT) pore sealing agents (cyclosporine A, carnitine and trifluoperazine), NADPH P_450_ reductase inhibitor (Diphenyliodonium chloride), CYP2E1 inhibitors (Phenylimidazole and 4-Methylpyrazole) and ATP generators (L-glutamine, Fructose and Xylitol)inhibitedcaspase-3 activation and cell death in algal lysate treated hepatocytes.Our data also confirmed that algal lysate activates apoptosis signaling via oxidative stress and mitochondrial pathway. Thus, ROS formation caused by the lysate exposure could directly be involved in mitochondrial MPT pore opening and activation of caspase-3 leading to *C.polykrikoides *lysateinduced apoptosis on rat hepatocytes. These findings contribute to a better understanding of *C.polykrikoides*-toxic effects on mammalian liver cells.

## Introduction

Harmful algal blooms (HABs) have become a significant threat to fisheries, public health, and economies around the world and have increased in frequency, duration, and distribution in recent decades. Most of documented HABs are caused by dinoflagellates and, under bloom conditions they can discolor affected waters, poison humans and marine animals, and disrupt aquatic ecosystems (1). 

A prolonged unusual HAB (about 10 months) started in August 2008 near the Strait of Hormuz and remained untill May 2009 on the Iranian side (2)of the Persian Gulf, and *Cochlodinium polykrikoides* was the causative agent (3).

*C. Polykrikoides *has been implicated in mass killing of wild and impounded fish around the globe (4-13). The fish killing activity of *C. Polykrikoides *has been well documented. Two major mechanisms already mentioned for *C. polykrikoides *ichthyotoxicity. Some scientist demonstrated that reactive oxygen species (ROS; *i.e*. superoxide anions and hydrogen peroxide) produced by *C. polykrikoides *cells may be one of the factors responsible for fish killing (7, 9, 11, 13, 14). Others suggested that multiple biologically active metabolites secreted by *C. polykrikoides *such as cytotoxic agents and mucus substances may contribute to the ichthyotoxicity of *C. polykrikoides*(10, 15, 16).

There are some reports that have showed *C. polykrikoides *not only contribute in mass mortality of fish, but also contribute to death of marine mammals(17-19).

There is only one document in the literature that reported *C. polykrikoides*lysate could be toxic in human erythrocytes (6). The same document reportedthat a relatively high concentration of *C. Polykrikoides*(5.27 × 10^6^ cells L^-1^) as the EC50 for its hemolytic activity on human erythrocytes. Unfortunately, there is a significant shortage of knowledge regarding any toxicity of this dinoflagellate on humans or even mammals. That is why we decided to plan this study to investigate probable toxic effect of *C. polykrikoides*lysate on rat liver hepatocytes. Since drinking contaminated water and feeding on contaminated fish are common ways of mammal(especially human’s) exposure to this harmful algae, thus liver could be a major target for *C. polykrikoides *possible toxicity in mammals.

In our overall screening researchwe used accelerated cytotoxicity mechanism screening (ACMS) methodsto determine the cytotoxic mechanisms of *C. polykrikoides*on rat hepatocytes.ACMSis describedin materials and methods.The ACMS usual parameters of toxicity include cell lysis (cytotoxicity marker), reactive oxygen species (ROS) formation (oxidative stress marker), glutathione (GSH) depletion (cellular antioxidant system marker), mitochondrial membrane potential decline (mitochondrial damage marker), ATP/ADP ratio (cellular energy depletion marker), cytochrome c release (starting point of apoptosis signaling), caspase 3 (final mediator of apoptosis) and finally apoptotic and necrotic phenotype detection.

## Experimental

*Chemicals*

Rhodamine 123, collagenase (from Clostridium histolyticum), bovine serum albumin, N-(2-hydroxyethyl)piperazine-N0-(2-ethanesulfonic acid) (HEPES), O-phthalaldehyde (OPT), reduced and oxidized glutathione (GSH and GSSG), 2,7-dichlorofluorescin diacetate (DCFH-DA), Trypan blue,GSe media, and heparin were purchased from Sigma- Aldrich Co. (Taufkrichen, Germany). All other chemicals were of the highest commercial grade available.

*Animals*

Male Sprague–Dawley rats (280–300 g) purchased from Pasteur Institute (Tehran, Iran), fed with a standard chow diet and water *ad libitum*, used for hepatocyte preparation. All experiments were conducted according to ethical standards and protocols approved by the Committee of Ethics, Shahid Beheshti University of Medical Sciences, Tehran, Iran.

*Accelerated cytotoxicity mechanism screening method*

This method determines the cytotoxic effectiveness of xenobiotics incubated for 6 h towards rat hepatocytes, freshly isolated from SD male rats. A functionomic approach is used to understand the cytotoxic mechanisms, *e.g*., the effects of inhibitors or protectants of cellular or sub-cellular damaging pathways on the loss of cell viability induced by the xenobiotic (*e.g*. algal lysate) are investigated. The procedures used are as follows:

(1) The concentration of xenobiotic (*e.g.*algal lysate) required for inducing a 50% loss of membrane integrity (EC_50_) of freshly isolated rat hepatocytes is determined by trypan blue exclusion.

(2) A major assumption with accelerated cytotoxicity mechanism screening (ACMS) is that high dose/short time (*in-vitro*) simulates low dose/long time (*in-vivo*) with relevance to human environmental exposure (20). The hepatocyte molecular cytotoxic mechanism of the xenobiotic (*e.g. *algal lysate) is determined by the changes in bioenergetics (ATP, mitochondrial membrane potential, *etc *…), oxidative stress (reduced/oxidized glutathione (GSH/GSSG), reactive oxygen species formation and *etc*…). If oxidative stress caused the cytotoxicity, then oxidative stress should precede cytotoxicity and antioxidants or ROS scavengers or redox therapy should prevent or delay the cytotoxicity. If not, then the oxidative stress likely occurred as a secondary result of the cytotoxicity. If mitochondrial toxicity caused the cytotoxicity, then glycolytic substrates should be protected and the membrane potential should be restored (20).

Antioxidants (*α*-tocopherol succinate and BHT), radical scavengers (mannitol and DMSO), mitochondrial permeability transition (MPT) pore sealing agents (cyclosporine A, carnitine and trifluoperazine), NADPH P_450_ reductase inhibitor (Diphenyliodonium chloride), CYP2E1 inhibitors (Phenylimidazole and 4-Methylpyrazole) and ATP generators (L-glutamine, Fructose and Xylitol) were used as protective agents in their sub-toxic concentrations in all our experiments. The basis for concentration selection for abovementioned preventing agents was previously published literature regarding the similar (ACMS) works performed in exactly similar technical conditions.

*Preparation of C. polykrikoides lysate*

A cell-pellet (about 120,000 cells), including a small amount of GSe media, was obtained from 30 mL of *C. polykrikoides *at late exponential growth phase (4 × 10^3^ cells/mL) by centrifugation at 5000×g for 5 min at 4°C, and was ruptured by ultrasonic treatment at 20°C in a bath-type sonicator. Microscopic observation confirmed that all *C. polykrikoides *cells wereruptured by this treatment. Lysate preparation of *C. polykrikoides *cells was achievedin a sonication bath for 1 min, repeated three times. The three lysates were combined, decanted and centrifuged at 3200×g for 15 min. The supernatant (total algal lysate) was removed and used for the cytotoxicity assays (modified from (16, 21).

The dinoflagellate concentration equivalent to obtained algal lysate was about 2.4 × 10^4^ cells/mL. considering that the lysate at 100% was obtained from *C. polykrikoides *cells.

*Isolation and incubation of hepatocytes*

Hepatocytes were obtained by collagenase perfusion of Sprague-Dawley rat liver (22), and viability was assessed by plasma membrane disruption determined by trypan blue (0.2 w/v) exclusion test. Cells were suspended at a density of 10^6^ cells/mL in round-bottomed flasks rotating in a water bath maintained at 37°C in Krebs–Henseleit buffer (pH = 7.4), supplemented with 12.5 mM HEPES under the atmosphere of 10% O_2_, 85% N_2_, and 5% CO_2_.

Each flask contained 10 mL of hepatocyte suspension. Hepatocytes were pre incubated for 30 min prior to the addition of chemicals. Stock solutions of all chemicals (×100 concentrated for the water solutions or ×1000 concentrated for the methanolic solutions) were prepared fresh prior to use. To avoid either non-toxic or severe toxic conditions in this study, EC50 concentrations were used for algae lysate. The EC50 of a chemical in hepatocyte cytotoxicity assessment technique (with the total 6 h incubation period) is defined as the concentration, which decreased the hepatocyte viability to 50% following the 3 h incubation period (23). In order to determine this value for algal lysate, concentration-response curves were plotted and then EC50 were determined based on a regression plot of three different concentrations (data and curves not shown). The EC503h concentration (*i.e*., 50% membrane lysis in 3 h) found for algal lysate was equivalent to 240 cells/mL. To incubate water soluble treatments with the required concentration, 100 μL sample of concentrated stock solution (×100 concentrated) was added to the rotating flask containing 10 mL of hepatocyte suspension (24-26). For the chemicals, which dissolved in methanol, methanolic stock solutions (×1000 concentrated) were prepared and to achieve the required concentration in the hepatocytes, 10 μL samples of stock solution was added to the 10 mL cell suspension(27). Ten micro liters of methanol did not affect hepatocyte viability after 6 h incubation (data not shown).

*Cell viability*

The trypan blue (0.2% w/v) exclusion test was used to determine the number of viable cells present in the cell suspension. It is based on the principle that live cells possess intact cell membranes that exclude trypan blue, whereas dead cells do not. In this test, a cell suspension was simply mixed with dye and then visually examined to determine whether cells take up or exclude dye. In the protocol, viable cells had clear cytoplasm whereas nonviable cells had blue cytoplasm (28). Aliquots of the hepatocyte incubate were taken at different time points during the 6 h incubation period. At least 80–90% of the control cells were still viable after 6 h.

*Determination of reactive oxygen species (ROS) formation*

Hepatocyte ROS generation induced by *C. polykrikoides*lysate was determined byadding dichlorofluorescin diacetate (DCFD) to the hepatocyte incubate. DCFD penetrates hepatocytes and ishydrolyzed to form non-fluorescent dichlorofluorescin. Dichlorofluorescin then reacts with ‘ROS’ to form the highly fluorescent dichlorofluorescein and effluxes the cell. ROS formation was assayed by withdrawing three-milliliter samples at different time points from *C. polykrikoides*lysate treated and control hepatocytes. These samples were then centrifuged for 1 min at 50×g. The cell pellet was then resuspended in three-milliliter incubation buffer containing 1.6 μM DCFD (29). The cells were then allowed to incubate in a thermostatic bath for 10 min with gentle shaking at 37°C. The fluorescence intensity of ROS product was measured at 500nm excitation and 520 nm emission wavelengths, using Hitachi F-2500 fluorescence spectrophotometer.

*Measurement of reduced and oxidized glutathione*

We measured the concentration of the reduced (GSH) and oxidized (GSSG) glutathione by a spectrofluorometric method (30). In order to confirm the linearity of the reaction rate in the adopted method, we used commercially purified GSH and GSSG to calibrate the standard curve. For GSH measurement, the final reaction mixture volume was 200 μL, which contained 180 μL of phosphate– EDTA buffer (0.1 M sodium phosphate–0.005 M EDTA, pH = 8.0), 10 μL of o-Phthalaldehyde (OPT, 100 μg per 100 μL methanol) and 10 μL of diluted sample (1:10 in phosphate–EDTA buffer). The reaction mixture was incubated for 15 min at room temperature, and the fluorescence was measured at excitation and emission wavelength of 350 nm and 450 nm, respectively using Hitachi F-2500 fluorescence spectrophotometer.The GSH content was expressed as μM per 10^6^cells/mL. GSSG was then measured using the same method outlined above, except that the sample was diluted with 10 volumes of 0.1 N NaOH containing 0.04 M of N-ethylmaleimide, instead of phosphate– EDTA buffer, in order to prevent the further oxidation of GSH to GSSG the pH of solution was adjustedto 12. The GSSG content was also expressed as μM per 106 cells/mL.

*Mitochondrial membrane potential assay*

The uptake and retention of the cationic fluorescent dye, rhodamine 123, has been used for the estimation of mitochondrial membrane potential. This assay is based on the fact that rhodamine 123 accumulates selectively in the mitochondria by facilitated diffusion. However, when the mitochondrial potential is decreased, the amount of rhodamine 123 that enters the mitochondria is also decreased as there is no facilitated diffusion. Thus, the amount of rhodamine 123 in the supernatant is increased and the amount in the pellet is decreased. Samples (500 μL) were taken from the cell suspension incubated at 37°C at different time points, and centrifuged at 50×g for 1 min. The cell pellet was then resuspended in 2 mL of fresh incubation medium containing 1.5 μM rhodamine 123 and incubated at 37°C in a thermostatic bath for 10 min with gentle shaking. Hepatocytes were separated by centrifugation and the amount of rhodamine 123 appearing in the incubation medium was measured fluorimetrically using Hitachi F-2500 fluorescence spectrophotometer set at 490 nm excitation and 520 nm emission wavelengths. The capacity of mitochondria to take up the rhodamine 123 was calculated as the difference in fluorescence intensity between control and treated cells (31).

*Assay of ATP and ADP*

ATP was measured based on bioluminescence generated from luciferin–luciferase reaction(32). The reaction seen below, results in the generation of measurable light at a wavelength of 562 nm:

ATP + Luciferin + O_2_^Luciferase^ Oxyluciferin + AMP + PP + CO + Light (562 nm)

Both intracellular ATP and ADP measurements were performed on rat hepatocytes using the standard curve of ATP. Light emission was measured with a Sirius tube luminometer, Berthold defection system (Germany). After calibration against the ATP standard, the ATP content of the cell lysate was determined. Then, ATP/ADP ratio was calculated.

In brief, the hepatocyte cells (about 10^6^ cells) were pelleted in a microcentrifuge tube by centrifugation at 3000×g for 10 min. The cellular ATP was then lysateed by adding 0.5 ml water and boiling the cell pellet for 5 min. The sample was then vortexed and centrifuged (3000×g for 5 min at 4°C), 12 μL of the supernatant was used for bioluminescence measurement. 12 μl of the supernatant was added to 25 μL cocktail (luciferin and Mg) and 10 μL of tris buffer. 5 μL luciferase enzyme was added to the mixture and luminescence was read at 562 nm. Luminescence intensity correlated to ATP level in hepatocytes. For total ATP measuring, 12 μL of the supernatant was added to 5 μL MgCl_2_ and 5 μL phosphoenol pyruvate and 12 μL phosphokinase. Phosphokinase changed the ADP to ATP. The mixture was incubated for 2 min and then 25 μL cocktail (luciferin and Mg) and 5 μL luciferase were added to the mixture and consequentlyluminescence was read at 562 nm. ADP level was calculated by calculating of difference between total ATP and primary ATP (33).

*Determination of cytochrome c release*

Cytochrome c release was determined by cytochrome c ELISA Kit (Quantikine M., R&D Systems, Abingdon, UK) according to the manufacturer’s instructions. In brief, cells were washed three times in PBS and then were re-suspended in Cell Lysis Buffer to a concentration of 1.5 x 10^6^ cells/mL. Cells were incubated for 1 h at room temperature with gentle mixing, and then the cells were centrifuged at 1000×g for 15 min. Supernatant was used for determination of cytochrome C. 

100 μL of calibrator diluent RD5P was added to each well of a 96 well plate. Then 100 μl of standard, control and sample were added per well. After covering plate with an adhesive strip, the plate was incubated for 2 h at room temperature (20-25°C). After 2 h incubation, wells were washed four times with washing buffer. After dryness, 200 μL of cytochrome c conjugate was added to each well and the plate was incubated for 2 h at room temperature. Then the wells were washed four times with washing buffer and 200 μL of substrate solution was added to each well and again plate was incubated for 30 min at room temperature with protection from light. Next 50 μL of stop solution was added to each well. Optical density of each well was finally determined within 30 min, using a microplate reader set to λ_max_= 450 nm, using Tecan-spectra ELISAreader.

*Determination of caspase-3 activity*

Caspase-3 activity was determined in cell lysate of hepatocytes by ‘‘Sigma’s caspase-3 assay kit (CASP-3-C)’’(34). In brief, this colorimetric assay is based on the hydrolysis of substrate peptide, Ac-DEVD-pNA, by caspase-3. The released moiety (p-nitroaniline) has a high absorbance at 405 nm. The concentration of the p-nitroaniline (μM) released from the substrate is calculated from the absorbance values obtained at λmax405 nm and then a calibration curve prepared with defined p-nitroaniline concentrations.

1X Assay Buffer was added to each of the wells. The Caspase 3 inhibitor was added to the appropriate wells. The reaction was started by adding 10 μL of caspase 3 substrate to each well and the well contents were then mixed gently by shaking to avoid bubble formation. Then the plate was covered and incubated at 37°C for 90 min. Absorbance of each well content was read at λmax405 nm. The results were calculated by a p-nitroaniline calibration curve, using Tecan-spectra ELISAreader.

*Detection of apoptosis (Annexin V-Cy3.18 binding assay)*

Apoptosis phenotype was detected using Sigma–Aldrich apoptosis detection kit. Detection of apoptosis was carried out as suggested by manufacturer’s kit protocol. Briefly, the cells were washed twice with buffer IV (the buffer that isolated hepatocytes were suspended in it) and suspended at a concentration of 1×10^6^ cellsmL^-1^. A circle of 1 cm diameter was drawn on a polyprep poly-L-lysine coated slide for each treatment. About 50 mL of cell suspension of each treatment was added to the circle of different slides and left at room temperature for 10 min. Cells were washed twice with the binding buffer and were stained with double label staining solution containing Annexin V-Cy3.18 (AnnCy3) and 6-carboxyfluorescein diacetate (6-CFDA). After washing with five aliquots of binding buffer, each circle was covered with a cover slip and visualized under a fluorescent microscope (Motic-AE31). In hepatocytes incubated with AnnCy3 and 6-CFDA simultaneously, live cells were labeled with 6-carboxyfluororescein (6-CF) (green), while necrotic cells only with AnnCy3 (red). However, the hepatocytes in the early stage of apoptosis were dyed with both AnnCy3 (red) spots within 6-CF (green) background. A total of 250 cells were counted, and were categorized as non-apoptotic, apoptotic, and necrotic cells based on the staining pattern and were expressed as percentage of cells.

*Statistical analysis*

Levene’s test was used to check the homogeneity of variances. Data were analyzed using one-way analysis of variance followed by Tukey’s HSD as the post-hoc test. Results were presented as mean ± SD of triplicate samples. The minimal level of significance chosen was p < 0.05.

## Results

Hepatocyte membrane lysiswas determined by trypan blue exclusion test. The EC50_3h_ concentration (*i.e*. 50% membrane lysis in 3 h) found for algal lysate was equivalent to 240 cells/mL. As shown in Table 1 algal lysatesignificantly increased hepatocyte cytotoxicity (hepatocyte membrane lysis) compared tocontrol hepatocytes (p < 0.05).When hepatocytes were incubated with algal lysate at this EC50_3h_ concentration, ROS formation determined by the oxidation of DCFH-DA to DCF was significantly increased (Table 2).

**Table 1:** Effect of antioxidants, ROS scavengers, MPT pore sealingagents, CYT P_450_ inhibitors and ATP generators on Algal extract inducedhepatotoxicity

**Cytotoxicity (%) 4h**	**Cytotoxicity (%) 3h**	**Cytotoxicity (%) 2h**	**Addition**
20 ± 3	18 ± 3	14 ± 2	Control rat hepatocytes
67 ± 5^a^	51 ± 4^a^	36 ± 3^a^	Algal extract (Eq. 240 cells/mL)
32 ± 3^b^	26 ± 3^b^	19 ± 2^b^	+*α*-Tocopherol succinate (10 μM)
33 ± 4^b^	24 ± 3^b^	21 ± 2^b^	+Butylatedhydroxytoluene (50 μM)
36 ± 4^b^	24 ± 2^b^	16 ± 2^b^	+Mannitol (50 mM)
40 ± 4^b^	31 ± 4^b^	26 ± 3^b^	+Dimethyl sulfoxide (150 μM)
49 ± 5^b^	40 ± 4^b^	28 ± 2^b^	+Cyclosporine A (2 mM)
53 ±5^b^	42 ± 4^b^	29 ± 2^b^	+Carnitine (2 mM)
53 ± 6b	39 ± 3^b^	28 ± 3^b^	+Trifluoprazine (15 μM)
45 ± 5^b^	33 ± 4^b^	18 ± 2^b^	+Fructose (10 mM)
44 ± 4^b^	35 ± 3^b^	16 ± 2^b^	+Xylitol (10 mM)
39 ± 4^b^	29 ± 3^b^	17±1^b^	+L-Glutamine (1 mM)
45 ± 5^b^	42 ± 4^b^	26 ± 2^b^	+Phenylimidazole (300 μM)
48 ± 6^b^	39 ± 4^b^	25 ± 3^b^	+4-Methylpyrazole (500 μM)
48 ± 4^b^	39 ± 4^b^	27 ± 3^b^	+Diphenyleneiodonium (0.05 mM)

Hepatocytes (10^6^ cells/mL) were incubated in Krebs–Henseleit buffer pH 7.4 at 37◦C for 4.0 h following the addition of EC50_3h_ of Algal extract. The dinoflagellate concentration equivalent to algal extract considers that the extract at 100% was obtained from a lysate of *C. polykrikoides*. Cytotoxicity was determined as the percentage of cells that take up trypan blue (Pourahmad and O’Brien, 2000).

Values are expressed as mean ± SD of three separate experiments (n = 3).

a Significant difference in comparison with control hepatocytes (p < 0.05).

b Significant difference in comparison with Algal extract (treated hepatocytes (p < 0.05).

**Table 2:** Effect of antioxidants, ROS scavengers, MPT pore sealing agents,CYT P_450_ inhibitors and ATP generators on algal extract ROS formation

**DCF** **Incubation time**					**Addition**
180 min	120 min	90 min	60 min	30 min	
4167 ± 120	4033 ± 79	398 5± 127	3923 ± 143	3570 ± 135	Control rat hepatocytes
5678 ± 155^a^	6754 ± 213^a^	6809 ± 245^a^	6892 ± 170^a^	6232 ± 156^a^	Algal extract (Eq. 240 cells/ml)
4358 ± 56^b^	4474 ± 450^b^	4768 ± 176^b^	4707 ± 196^b^	4534 ± 102^b^	+α-Tocopherol succinate (10 μM)
4474 ± 84^b^	4506 ± 175^b^	4957 ± 98^b^	4854 ± 108^b^	4635 ± 86 ^b^	+Butylatedhydroxytoluene (50 μM)
4324 ± 166^b^	3464 ± 202^b^	4734 ± 110^b^	4455 ± 55^b^	4345 ± 207^b^	+Mannitol (50 mM)
4298 ± 203^b^	4132 ± 142^b^	4345 ± 143^b^	4326 ± 74^b^	4198 ± 99^b^	+Cyclosporine A (2 mM)
4148 ± 87^b^	3957 ± 131^b^	4786 ± 128^b^	4541 ± 91^b^	4693 ± 176^b^	+Carnitine (2 mM)
4324 ± 402^b^	4234 ± 185^b^	4602 ± 140^b^	3876 ± 152^b^	3822 ± 179^b^	+Trifluoprazine (15 μM)
3840 ± 75^b^	4168 ± 570^b^	3978 ± 149^b^	4276 ± 305^b^	3843 ± 430^b^	+Fructose (10 mM)
4376 ± 402^b^	4106 ± 185^b^	4906 ± 140^b^	3957 ± 152^b^	3903 ± 179^b^	+Xylitol (10 mM)
4341 ± 41^b^	3764 ± 212^b^	4976 ± 151^b^	4843 ± 137^b^	4387 ± 570^b^	+L-Glutamine (1 mM)
3834 ± 102^b^	4324 ± 462^b^	3876 ± 176^b^	4243 ± 312^b^	3876 ± 421^b^	+Phenylimidazole (300 μM)
3780 ± 232^b^	4235 ± 402^b^	402 1± 215^b^	4324 ± 412^b^	3934 ± 376^b^	+4-Methylpyrazole (500 μM)
4427 ± 402^b^	4173 ± 207^b^	4592 ± 137^b^	3972 ± 125^b^	3745 ± 187^b^	+Diphenyleneiodonium (0.05 mM)

Hepatocytes (10^6^ cells/mL) were incubated in Krebs–Henseleit buffer pH 7.4 at 37^◦^C for 3.0 h following the addition of EC50_3h_ of Algal extract. The dinoflagellate concentration equivalent to algal extract considers that the extract at 100% was obtained from a lysate of *C. polykrikoides*. DCF formation was expressed as fluorescent intensity units (pourahmad *et al.*, 2009).

Values are expressed as mean ± SD of three separate experiments (n = 3).

a Significant difference in comparison with control hepatocytes (p < 0.05).

b Significant difference in comparison with Algal extract (treated hepatocytes (p < 0.05).

 Algallysate-induced cytotoxicity and ROS generation was prevented by antioxidants (*α*-tocopherol succinate and BHT), radical scavengers (mannitol and DMSO), NADPH P_450_ reductase inhibitor (Diphenyliodonium chloride), CYP2E1 inhibitors (Phenylimidazole and 4-Methylpyrazole), MPT pore sealing agents (cyclosporine A, carnitine and trifluoperazine), glycolytic ATP generators (fructose and xylitol), and mitochondrial ATP generator (L-glutamine) (Table 2). All of these reagents did not produce any marked alterations in cytotoxicity and ROS formation at concentrations used (data not shown).

One of the earliest intracellular events that usually occurs upon initiation of apoptosis is disruption of mitochondrial transmembrane potential. As shown in Table 3, algal lysate induced a rapid decline of mitochondrial membrane potential in hepatocytes which were prevented by antioxidants (*α*-tocopherol succinate and BHT), radical scavengers (mannitol and DMSO), NADPH P_450_ reductase inhibitor (Diphenyliodonium chloride), CYP2E1 inhibitors (Phenylimidazole and 4-Methylpyrazole) and both glycolytic and mitochondrial ATP generators (fructose, xylitol, and L-glutamine). All of these reagents did not produce any marked changes in hepatocyte mitochondrial membrane potential at concentrations used (data not shown).

**Table 3:** Preventing of mitochondrial membrane potential decline induced by algal extract by antioxidants, ROS scavengers, MPT pore sealing agents, CYT P_450_ inhibitors and ATP generators

**%ΔΨm** **Incubation time**						**Addition**
180 min	120 min	**90 min**		**60 min**	**30 min**	
5 ± 1	4 ± 1		3 ± 1	2 ± 1	2 ± 1	Control rat hepatocytes
28 ± 2^a^	20 ± 2^a^		16 ± 2 ^a^	19 ± 1 ^a^	13 ± 1 ^a^	Algal extract (Eq. 240 cells/ml)
12 ± 1^b^	12 ± 2^b^		10 ± 2^b^	5 ± 1^b^	6 ± 1^b^	+α-Tocopherol succinate (10 μM)
12 ± 1^b^	11 ± 2^b^		8 ± 2^b^	6 ± 1^b^	5 ± 1^b^	+Butylatedhydroxytoluene (50 μM)
7 ± 1^b^	6 ± 1^b^		6 ± 2^b^	8 ± 1^b^	5 ± 1^b^	+Mannitol (50mM)
7 ± 1^b^	6 ± 1^b^		6 ± 2^b^	8 ± 1^b^	5 ± 1^b^	+Dimethyl sulfoxide (150 μM)
7 ± 1^b^	6 ± 1^b^		6 ± 2^b^	8 ± 1^b^	5 ± 1^b^	+Cyclosporine A (2 mM)
15 ± 1^b^	12 ± 2^b^		10 ± 2^b^	8 ± 1^b^	2 ± 1^b^	+Carnitine (2 mM)
14 ± 1^b^	13 ± 1^b^		9 ± 2^b^	7 ± 1^b^	6 ± 1^b^	+Trifluoprazine (15 μM)
16 ± 1^b^	13 ± 1^b^		11 ± 2^b^	8 ± 1^b^	7 ± 1^b^	+Fructose (10 mM)
14 ± 1^b^	13 ± 1^b^		9 ± 2^b^	7 ± 1^b^	6 ± 1^b^	+Xylitol (10 mM)
6 ± 1^b^	4 ± 2^b^		5 ± 1^b^	3 ± 1^b^	3 ± 1^b^	+L-Glutamine (1 mM)
6 ± 1^b^	4 ± 2^b^		5 ± 1^b^	3 ± 1^b^	3 ± 1^b^	+Phenylimidazole (300 μM)
6 ± 2^b^	5 ± 2^b^		7 ± 1^b^	4 ± 1^b^	2 ± 1^b^	+4-Methylpyrazole (500 μM)
10 ± 1^b^	11 ± 2^b^		7 ± 1^b^	4 ± 1^b^	2 ± 1^b^	+Diphenyleneiodonium (0.05 mM)

Hepatocytes (10^6^ cells/mL) were incubated in Krebs–Henseleit buffer pH 7.4 at 37◦C for 3.0 h following the

addition of EC50_3h_ of Algal extract. The dinoflagellate concentration equivalent to algal extract considers that the extract at 100% was obtained from a lysate of C. polykrikoides. Mitochondrial membrane potential was determined as the difference in mitochondrial uptake of the rhodamine 123 between control and treated cells and expressed as fluorescence intensity unit (Andersson et al., 1987).

Values are expressed as mean ± SD of three separate experiments (n = 3).

aSignificant difference in comparison with control hepatocytes (p < 0.05).

bSignificant difference in comparison with Algal extract (treated hepatocytes (p < 0.05).

All the percentage data were transformed after statistical analysis.

As shown in Table 4, incubation of hepatocytes with algal lysate caused rapid hepatocyte GSH depletion. Most of the algal lysate-induced hepatocyte GSH depletion could be attributed to the expulsion of GSSG (Table 4). Again antioxidants (*α*-tocopherol succinate and BHT), radical scavengers (mannitol and DMSO), mitochondrial MPT pore sealing agents (cyclosporine A, carnitine and trifluoperazine), NADPH P_450_ reductase inhibitor (Diphenyliodonium chloride), CYP2E1 inhibitors (Phenylimidazole and 4-Methylpyrazole) and both glycolytic and mitochondrial ATP generators (fructose, xylitol, and L-glutamine) significantly (p < 0.05) prevented both algal lysate induced intracellular GSH decrease and extracellular GSSG increase (Table 4). All of these reagents did not show any significant effect on hepatocytes GSH/GSSG status at concentrations used (data not shown).

**Table 4:** Effect of antioxidants, ROS scavengers, MPT pore sealingagents, CYT P_450_ inhibitors and ATP generators on Algal extract inducedglutathione depletion

**Extra cellular** **GSSG (μM)** **90 min**	**Intracellular GSH (μM)** **90 min**	**Addition**
5.1 ± 0.5	49 ± 4	Control rat hepatocytes
11 ± 1^a^	13 ± 1^a^	Algal extract (Eq. 240 cells/mL)
4 ± 0.4^b^	37 ± 3^b^	+*α*-Tocopherol succinate (10 μM)
4 ± 0.5^b^	39 ± 3^b^	+Butylatedhydroxytoluene (50 μM)
5.5 ± 0.5^b^	28 ± 5^b^	+Mannitol (50 mM)
5.5 ± 0.6^b^	27 ± 3^b^	+Dimethyl sulfoxide (150 μM)
4.5 ± 0.4^b^	24 ± 3^b^	+Cyclosporine A (2 mM)
5 ± 0.5^b^	25 ± 2^b^	+Carnitine (2 mM)
5.6 ± 0.7^b^	30 ± 3^b^	+Trifluoprazine (15 μM)
3.6 ± 0.3^b^	26 ± 3^b^	+Fructose (10 mM)
6 ± 0.6^b^	25 ± 2^b^	+Xylitol (10 mM)
4.8 ± 0.4^b^	35 ± 3^b^	+L-Glutamine (1 mM)
4.7 ± 0.5^b^	28 ± 4^b^	+Phenylimidazole (300 μM)
4.9 ± 0.6^b^	25 ± 3^b^	+4-Methylpyrazole (500 μM)
6.4 ± 0.6^b^	36 ± 5^b^	+Diphenyleneiodonium (0.05 mM)

Hepatocytes (10^6^ cells/mL) were incubated in Krebs–Henseleit buffer pH 7.4 at 37^◦^C for 90 min following the addition of EC50_3h_ of Algal extract. The dinoflagellate concentration equivalent to algal extract considers that the extract at 100% was obtained from a lysate of C. polykrikoides. Intracellular GSH and extra cellular GSSG were flurimetrically determined as described byHissin and Hilf, 1978.

Values are expressed as mean ± SD of three separate experiments (n = 3).

^a^Significant difference in comparison with control hepatocytes (p < 0.05).

^b^Significant difference in comparison with Algal extract (treated hepatocytes (p < 0.05).

As shown in Table 5, algal lysate decreased ATP level and also increased ADP/ATP ratio in hepatocytes. Again antioxidants antioxidants (*α*-tocopherol succinate and BHT), radical scavengers (mannitoland DMSO), mitochondrial MPT pore sealing agents (cyclosporine A, carnitine and trifluoperazine), NADPH P_450_ reductase inhibitor (Diphenyliodonium chloride), CYP2E1 inhibitors (Phenylimidazole and 4-Methylpyrazole) and both glycolytic and mitochondrial ATP generators (fructose, xylitol, and L-glutamine) significantly (p < 0.05) prevented both algal lysate induced ATP level decrease and ADP/ATP ratio increase in hepatocytes (Table 5).All of these reagents did not show anytoxic effect on ATP isolated rat hepatocytesat concentrations used (data not shown).

**Table 5:** ATP level andADP/ATP ratio changes induced by algae extract and effect of antioxidants, ROS scavengers, MPT pore sealing agents, CYT P_450 _inhibitorsand ATP generators on ADP/ATP ratio

**ADP/ATP ratio**	**ATP 120 min**	**Addition**
0.19	65.1 ± 3.5	Control rat hepatocytes
0.71	41.3 ± 1.9^a^	Algal extract (Eq. 240 cells/mL)
0.41	54.6 ± 2.6^b^	+*α*-Tocopherol succinate (10 μM)
0.36	57.3 ± 2.4^b^	+Butylatedhydroxytoluene (50 μM)
0.34	55.5 ± 2.2^b^	+Mannitol (50 mM)
0.38	53.7 ± 3.4^b^	+Dimethyl sulfoxide (150 μM)
0.30	58.9 ± 2.9^b^	+Cyclosporine A (2 mM)
0.29	48.4 ± 2.3^b^	+Carnitine (2 mM)
0.26	54.8 ± 3.1^b^	+Trifluoprazine (15 μM)
0.12	56.2 ± 3.3^b^	+Fructose (10 mM)
0.16	46.4 ± 2.6^b^	+Xylitol (10 mM)
0.09	48.1 ± 3.4^b^	+L-Glutamine (1 mM)
0.45	56.7 ± 3.2^b^	+Phenylimidazole (300 μM)
0.42	53.6 ± 2.9^b^	+4-Methylpyrazole (500 μM)
0.39	51.3 ± 2.8^b^	+Diphenyleneiodonium (0.05 mM)

Hepatocytes (10^6^ cells/mL) were incubated in Krebs–Henseleit buffer pH 7.4 at 37^◦^C for 120 min following the addition of EC50_3h_ of Algal extract. The dinoflagellate concentration equivalent to algal extract considers that the extract at 100% was obtained from a lysate of C. polykrikoides. The ratio of ADP:ATP was assessed using Bradbury method (Bradbury *et al*., 2000).

Values are expressed as mean ± SD of three separate experiments (n = 3).

^a^Significant difference in comparison with control hepatocytes (p < 0.05).

^b^Significant difference in comparison with Algal extract (treated hepatocytes (p < 0.05).

**Figure 1:**
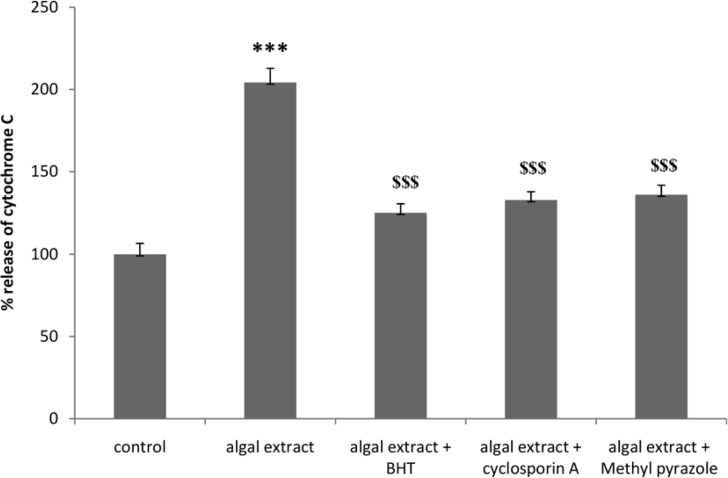
Effect of algal extract-induced cytochrome c release in isolated rat hepatocyte after 90 min exposure. Cytochrome c release was measured using an ELISA kit.Values are expressed as mean±SD of three separate experiments (n = 3).*: Significant difference in comparison with control hepatocytes (* p < 0.05; ** p < 0. 01; ***p < 0.001).$: Significant difference in comparison with Algal extract (**$ **p < 0.05; **$$ **p < 0. 01; **$$$ **p < 0.001).

Induction of mitochondrial permeability transition (MPT) is supposed to be the resultof the opening of the related pores in mitochondrial membrane leading to the release of cytochrome C (35). In order to confirm the occurrence of this intra-mitochondrial protein release in to the cytosol, cytochrome c release in algal lysate treated rat hepatocyte was determined spectrophotometrically at λ_max_ 450 nm with an ELISA reader. As shown in Figure 1, algal lysateinduced cytochrome c release following 1 h of incubation in isolated rat hepatocyte before cytotoxicity ensued. Algal lysate induced cytochrome c release was prevented by MPT pore sealing agent (cyclosporine A), antioxidant (BHT) and CYP2E1 inhibitor (4-methylpyrazole). Cyclosporine A, BHT and 4-methylpyrazole did not cause any significant effect on the hepatocyte mitochondrial cytochrome C release at concentrations used (data not shown).

Algal lysate also increased apoptosis signaling final mediator caspase-3 activity in isolated rat hepatocytes following 120 min of incubation (Table 6). Increased caspase-3 activity was prevented by antioxidants (*α*-tocopherol succinate and BHT), radical scavengers (mannitol and DMSO), mitochondrial MPT pore sealing agents (cyclosporine A, carnitine andtrifluoperazine), NADPH P_450_ reductase inhibitor (Diphenyliodonium chloride), CYP2E1 inhibitors (Phenylimidazole and 4-Methylpyrazole), and both glycolytic and mitochondrial ATP generators (fructose, xylitol, and L-glutamine) (Table 6). All of these compounds did not produce any marked changes in hepatocyte caspases-3 activity at the concentrations used (data not shown). 

**Figure 2:**
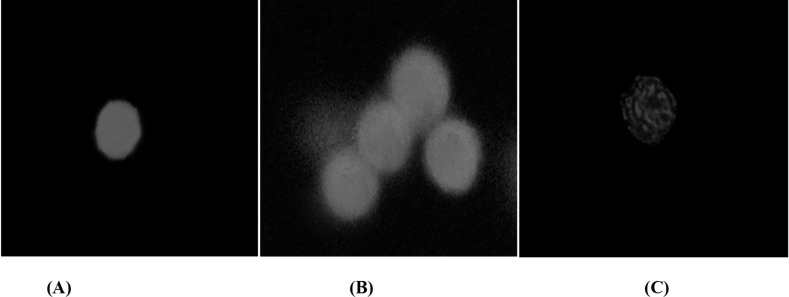
Apoptosis phenotype in rat hepatocytes following the exposure of algal extract detected by AnnCy3. 6-CFDA is a non-fluorescent compound and when enters a live viable cell (control cell), it is hydrolyzed and converted to a highly fluorescent compound 6-CF (green fluorescence) that remains in the cytoplasm (photograph A). After the apoptosis initiated, the membrane PS translocated from the inner leaflet of the plasma membrane to the cell surface. There, PS can be easily identified by staining with a red fluorescent conjugate of Annexin V. Hepatocytes in theearly stage of apoptosis will be dyed with both AnnCy3 (red) spots within 6-CF (green) background. Photograph B shows apoptosis phenotype in hepatocytes exposed to algal extract. Necrotic cells will only be labeled with AnnCy3 (red). Photograph C shows a necrotic cell

After initiation of apoptosis, phosphatidylserine (PS) gradually appears on the outer leaflet of the plasma membrane of hepatocytes due to inhibition of Mg-ATP dependent aminophospholipid translocase, which could be detected by Cy3 conjunct Annexin V, giving off red fluorescence. In the AnnCy3 binding assay, the hepatocytes were categorized as nonapoptotic (green), apoptotic (green and red), and necrotic (red). The hepatocytes that bind to Annexin V, which is representative of cells undergoing apoptosis was shown in Figure 2. Algal lysate at its EC50 concentrations showed significant increase in the number of apoptotic phenotype in the algal lysate treated rat hepatocytes compared to control cells (Table 7).

**Table 6:** Preventing algae extract-induced caspase-3 activation by antioxidants, ROS scavengers, MPT pore sealing agents, CYT P_450_ inhibitors and ATP generators

**Caspase-3 activity 120 min** **(μM pNA/mL/min)**	**Addition**
5 ± 1	Control rat hepatocytes
28 ± 2 ^a^	Algal extract (Eq. 240 cells/mL)
12 ± 1^b^	+*α*-Tocopherol succinate (10 μM)
12 ± 1^b^	+Butylatedhydroxytoluene (50 μM)
7 ± 1^b^	+Mannitol (50 mM)
16 ± 3^b^	+Dimethyl sulfoxide (150 μM)
7 ± 1^b^	+Cyclosporine A (2 mM)
15 ± 1^b^	+Carnitine (2 mM)
17 ± 2^b^	+Trifluoprazine (15 μM)
16 ± 1^b^	+Fructose (10 mM)
14 ± 1^b^	+Xylitol (10 mM)
13 ± 1^b^	+L-Glutamine (1 mM)
17 ± 3^b^	+Phenylimidazole (300 μM)
15 ± 2^b^	+4-Methylpyrazole (500 μM)
20 ± 2^b^	+Diphenyleneiodonium chloride (0.05 mM)

Hepatocytes (10^6^ cells/mL) were incubated in Krebs–Henseleit buffer pH 7.4 at 37^◦^C for 120 min following the addition of EC50_3h_ of Algal extract. The dinoflagellate concentration equivalent to algal extract considers that the extract at 100% was obtained from a lysate of C. polykrikoides. Caspase-3 activitywas determined by Sigma-Aldrich kit (Sakahira *et al*;1998).The kit determines produced pNA that is released from the interaction of caspase-3 and AC-DEVD-pNA (peptide substrate).

Values are expressed as mean ± SD of three separate experiments (n = 3).

^a^Significant difference in comparison with control hepatocytes (p < 0.05).

^b^Significant difference in comparison with Algal extract (treated hepatocytes (p < 0.05).

**Table 7:** Effects of algal extract on induction of apoptosis

**% Necrotic** **Hepatocytes** **180 min**	**% Apoptotic** **Hepatocytes** **180 min**	**% Non apoptotic-Necrotic** **Hepatocytes** **180 min**	**Addition**
17 ± 3	5 ± 3	78 ± 8	Control rat hepatocytes
24 ± 5^a^	29 ± 6^a^	47 ± 5^a^	Algal extract (Eq. 240 cells/mL)
25 ± 4^a^	32 ± 5^a^	43 ± 8^a^	Positive control (30 mM NaAsO2)

Hepatocytes (10^6^ cells/mL) were incubated in Krebs–Henseleit buffer pH 7.4 at 37^◦^C for 180 min following the addition of EC50_3h_ of Algal extract. The dinoflagellate concentration equivalent to algal extract considers that the extract at 100% was obtained from a lysate of C. polykrikoides. Following the addition of algal extract, apoptotic phenotypes were measured. Values show the percentage of cells that bind to Annexin V observed under fluorescent microscope, which were representatives of cells undergoing apoptosis or necrosis.

Values are expressed as mean_SD of three separate experiments (n = 3).

^a^Significant difference in comparison with control hepatocytes (p < 0.05).

## Discussion

Apoptosis is a highly organized process characterized by the progressive activation of precise pathways leading to specific biochemical and morphological alterations (23). Initial stages of apoptosis are characterized by alterations in the cellular redox potential, cell shrinkage, and loss of membrane lipid asymmetry (*e.g., *translocation of PS from the inner face of the plasma membrane to the cell surface). Later stages associated with the execution phase of apoptosis are characterized by activation of caspase-3 and endonucleases, apoptotic body formation, and cell fragmentation (36). Apoptosis is considered as the major form of chemical induced cell death while necrosis rarely occurs, it only happens only in circumstances of severe cell injury (37-39).

In this study, algal lysate significantly increased ROS formation following incubation in rat hepatocytes. Excessive ROS formation resulted in cellular redox imbalance, oxidative stress, and hepatocyte cell lysis. ROS can react with polyunsaturated fatty acids and generate lipid hydroperoxides and *α, ß*-unsaturated aldehydes (*e.g*. 4-hydroxy-2-nonenal) which are highly electrophilic, unstable, readily propagating between cellular compartments, and capable of reacting with proteins and nucleic acids (40). In the liver, this lipid peroxidation is associated with impairment of membrane-dependent functions of mitochondria (oxidative metabolism) and lysosomes (membrane integrity, fluidity, and pH). The excessive generation of ROS ultimately leads to apoptosis (41). It was shown that oxidative stress plays a central role in the induction of apoptotic pathways (42-47). The mitochondrion is known to be the major intracellular source of ROS production in cells (48, 49) and many toxins exert their pro-oxidant effects through a direct damage of mitochondria (50). In apoptosis, it is postulated that mitochondria are most important organelle in mediating apoptosis (41). Excess ROS oxidizes the thiol groups in the MPT pore regions of mitochondrial membrane leading to cross-linking of thiol groups in these regions; as a consequence, mitochondrial membrane undergoes conformational alterations, and mitochondrial MPT pore opens. Central to mitochondrial permeabilization and mitochondrial release of apoptogenic factors is the MPT pore, a megapore spanning the inner and outer mitochondrial membranes (51). Excess ROS also increase the mitochondrial membrane permeability and damage the respiratory chain resulting in even higher ROS production.

CYT _450_ isoenzymes, especially CYP2E1 are effective catalysts for ROS production and they are one the most powerful inducers of oxidative stress in liver cells (52). In our study algal lysate induced ROS formation was prevented by NADPH P_450_ reductase inhibitor (diphenyliodonium chloride),CYP2E1 inhibitors (1-phenylimidazole, and 4-methylpyrazole) and mitochondrial pore sealing agents (cyclosporine A, carnitine and trifluoperazine) suggesting that at least two different intracellular sources: cytochrome P_450_and mitochondrial electron transfer chain disruption are involved in algal lysate induced ROS formation and its subsequent cytotoxic events in rat hepatocytes.

Pre-incubation of NADPH P_450_ reductase inhibitor (diphenyliodonium chloride) and CYP2E1 inhibitors (1-phenylimidazole, and 4-methylpyrazole) also inhibited hepatocyte membrane lysis, glutathione depletion, collapse of mitochondrial membrane potential, ATP depletion and increase in ADP/ATP ratio, activation of caspase-3 and finallyappearance of apoptosis phenotype. All these protective effects suggest that CYT P_450_especially CYP 2E1 plays a very important role in metabolic activation of certain toxic compound in *Cochlodinium polykrikoides *algal lysate.

Glutathione (GSH) is a ubiquitous thiol-containing tripeptide, which plays a key role in cellular defense against xenobiotics and naturally occurring deleterious compounds such as free radicals and hydroperoxides. GSH is a highly sensitive indicator of cell functionality and viability. The overwhelming level of intracellular ROS and GSSG (oxidized form of GSH) indicate a disturbance in the Redox status of the cell, a condition that may be followed by apoptosis (53)*. *ROS could potentially be an apoptosis inducer because GSH depletion could induce apoptosis via altering the expression of the Bcl-2 family proteins and MPT pore opening caspases are sensitive to redox state of the cell; it was also shown that antioxidants could prevent apoptosis (54). Besides, in this study we realized that depleting hepatocyte’s most important antioxidant peptide, GSH, potentiated all algal lysateinduced loss of cell viability, ROS formation, mitochondrial membrane potential collapse (data not shown). Therefore, it is confirmed that the cytotoxic mechanism of algal lysate is mediated by oxidative stress.

In our study, both algal lysate-induced cytotoxicity and ROS generation were prevented by MPT pore sealing agents, and glycolytic ATP generators, fructose, xylitol (ATP is a pore sealing agent), indicating that the mitochondrial MPT pore opening is involved in algal lysate-induced toxicity and oxidative stress. Our results also showed that the hepatocyte mitochondrial membrane potential was rapidly decreased by algal lysate, which again was prevented by antioxidants and ROS scavenger indicating that mitochondrial membrane damage was a consequence of ROS formation. Furthermore, the ATP generators (fructose, xylitol, and L-glutamine) prevented algal lysate-induced mitochondrial membrane potential decrease, which indicates that the collapse may be a consequence of MPT pore opening and ATP depletion following the thiol cross-linking of the pore region. Therefore, the mechanism of algal lysate-induced mitochondrial damage seems to be a result of increased ROS formation in hepatocytes.

Growing evidence suggest that apoptosis is accompanied by opening of the mitochondrial permeability pores, leading to disruption of the mitochondrial transmembrane potential. This results in caspase activation due to the release of cytochrome C, ATP and apoptogenic factors into the cytosol. Several observations indicate that cellular ATP level is an important determinant for cell death, either by apoptosis or necrosis. It is proven that cells need a certain ATP level to stay alive, so if the amount of ATP falls below this level apoptosis or necrosis could occur (55, 56). Release of cytochrome c from mitochondria into cytosol is a key initiating step in both apoptotic and necrotic cell death processes in intact cells (35).There is a correlation between intracellular ATP level and cell proliferation. The percentage of viable cells correlated positively with the ATP values and negatively with the ADP: ATP ratio (57, 58).This is quite in accordance with our results, indicating consistent decrease of hepatocyte viability and ATP depletion (Table 5) and significant increase in caspases-3 activity, the final mediator of apoptosis.

The caspases are the most important effector molecules in the execution of apoptosis and progression of the caspase cascade activation ends in the activation of caspase-3, the finalmediator of apoptosis. In our study algal lysate induced release of cytochrome c from mitochondria into the cytosol following 1 hour of incubation in isolated rat hepatocyte before cytotoxicity ensued. Algal lysate induced cytochrome c release was prevented by MPT pore sealing agent (cyclosporine A), antioxidant (BHT) and CYP2E1 inhibitor (4-methylpyrazole) suggesting that *C. polykrikoides *induced cytochrome c expulsion was subsequent to MPT pore opening due to ROS formation probably resulting from CYP2E1 mediated bioactivation of algal components in liver hepatocytes. Caspase-3 activity was also increased in hepatocytes when incubated with algal lysate. Increased caspase-3 activity was prevented by antioxidants, radical scavengers, MPT pore sealing agents, and ATP generators indicating that the activation of caspase-3 was a consequence of ROS formation and mitochondrial MPT pore opening.

One of the cellular changes involved in apoptosis process is loss of cell membrane phospholipid asymmetry during early stages of apoptosis. In viable cells, phosphatidylserine (PS) is usually transported to the inner plasma membrane leaflet by the enzyme Mg-ATP dependent aminophospholipid translocase. However, during the onset of apoptosis, PS is gradually concentrated in the external leaflet of the plasma membrane due to enzyme inhibition. The PS is then available for binding to Annexin V and any of its fluorescent conjugates (59). The binding is observed as red fluorescence intensity. 6-CFDA is a non-fluorescent compound and when entering living cells, undergoes hydrolysis and oxidation of two electrons, respectively, and converts to a highly fluorescent compound, 6-carboxyfluororescein (6-CF) that emits a green fluorescence, visible under fluorescent microscope. Our results provided direct evidence that exposure to algal lysate could induce apoptosis phenotype in the hepatocytes. The apoptosis phenotype disappeared when algal lysate-treated hepatocytes were pre-incubated with antioxidants (*α*-tocopherol succinate and BHT), radical scavengers (mannitol and DMSO), MPT pore sealing agents (cyclosporine A, carnitine and trifluoperazine) and ATP generators (fructose, xylitol, and L-glutamine). This suggests that *C. polykrikoides *induces apoptosis in mammalian hepatocytes by generation of ROS and alteration in mitochondrial function. 

## Conclusion

Our results confirmed that algal lysate induced liver toxicity is resulted from different cytotoxic events including: increased ROS formation and subsequent change of mitochondrial membrane conformation to opened MPT pore position and cytochrome C release in to the cytosol which causes mitochondrial collapse of Ψm. Cytochrome C release is the regulating step for mitochondria-mediated cell death signaling which could lead to both apoptosis and necrosis.

To our knowledge, this is the first report that investigated the cytotoxicity of *Cochlodinium polykrikoides *in mammalian liver cells in detail and provided a complete mechanistic logic for *C. polykrikoides *toxicity in mammals.
